# 1-[(3-Methyl­piperidin-1-yl)(phen­yl)meth­yl]-2-naphthol

**DOI:** 10.1107/S1600536809017176

**Published:** 2009-05-14

**Authors:** Wen Xiang Wang, Hong Zhao

**Affiliations:** a Ordered Matter Science Research Center, College of Chemistry and Chemical Engineering, Southeast University, Nanjing 210096, People’s Republic of China

## Abstract

In the title compound, C_23_H_25_NO, the dihedral angle between the naphthyl­ene ring system and the benzene ring is 78.17 (10)°. The mol­ecular conformation is stabilized by a strong intra­molecular O—H⋯N hydrogen bond.

## Related literature

For the structures of related compounds, see: Szatmari & Fulop (2004 ▶); Zhao & Sun (2005 ▶); Wang & Zhao (2008 ▶); Wan & Zhao (2008 ▶).
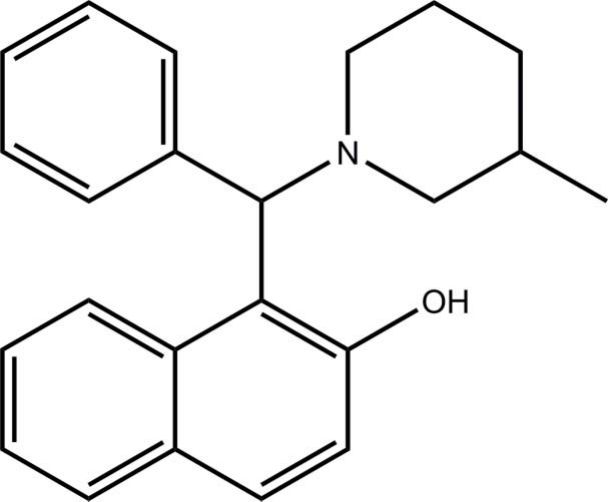


## Experimental

### 

#### Crystal data

C_23_H_25_NO
*M* *_r_* = 331.44
Monoclinic, 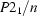

*a* = 9.2138 (13) Å
*b* = 10.9056 (13) Å
*c* = 18.635 (3) Å
β = 97.007 (10)°
*V* = 1858.5 (5) Å^3^
*Z* = 4
Mo *K*α radiation
μ = 0.07 mm^−1^
*T* = 292 K
0.30 × 0.25 × 0.20 mm

#### Data collection

Rigaku SCXmini diffractometer
Absorption correction: multi-scan (*CrystalClear*; Rigaku, 2005 ▶) *T*_min_ = 0.965, *T*_max_ = 0.979
19217 measured reflections
4422 independent reflections
2302 reflections with *I* > 2σ(*I*)
*R*_int_ = 0.067

#### Refinement

*R*[*F* ^2^ > 2σ(*F* ^2^)] = 0.061
*wR*(*F* ^2^) = 0.155
*S* = 0.99
4422 reflections
228 parameters
H-atom parameters constrained
Δρ_max_ = 0.15 e Å^−3^
Δρ_min_ = −0.16 e Å^−3^

### 

Data collection: *CrystalClear* (Rigaku, 2005 ▶); cell refinement: *CrystalClear*; data reduction: *CrystalClear*; program(s) used to solve structure: *SHELXS97* (Sheldrick, 2008 ▶); program(s) used to refine structure: *SHELXL97* (Sheldrick, 2008 ▶); molecular graphics: *SHELXTL/PC* (Sheldrick, 2008 ▶); software used to prepare material for publication: *SHELXTL/PC*.

## Supplementary Material

Crystal structure: contains datablocks I, global. DOI: 10.1107/S1600536809017176/rz2319sup1.cif

Structure factors: contains datablocks I. DOI: 10.1107/S1600536809017176/rz2319Isup2.hkl

Additional supplementary materials:  crystallographic information; 3D view; checkCIF report

## Figures and Tables

**Table 1:** Hydrogen-bond geometry (Å, °)

*D*—H⋯*A*	*D*—H	H⋯*A*	*D*⋯*A*	*D*—H⋯*A*
O1—H1*A*⋯N1	0.82	1.84	2.570 (2)	148

**Fig. 1.**
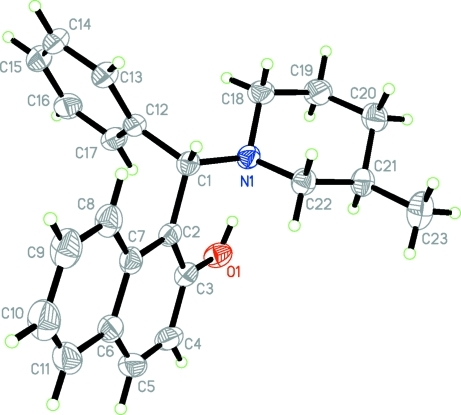
The structure of the title compound, showing the atom-numbering scheme. Displacement ellipsoids are drawn at the 30% probability level.

## supplementary crystallographic information

### Comment

It is well known that many compounds derived from naphthalen-2-ol have received much attention in organic chemistry (Szatmari & Fulop, 2004; Zhao & Sun, 2005). Recently, we reported the synthesis and crystal structures of 1-[(dimethylamino)(phenyl)methyl]naphthalen-2-ol (Wang & Zhao, 2008) and 1-[pyrrolidin-1-yl(*p*-tolyl)methyl]naphthalen-2-ol (Wan & Zhao, 2008). We now report the crystal structure of the title compound.

Bond lengths and angles in the title compound have normal values. The dihedral angle between the naphthyl and phenyl rings is 78.17 (10)°. The molecular conformation is stabilized by a strong intramolecular O—H···N hydrogen bond (Table 1). The crystal packing is mainly stabilized by van der Waals interactions.

### Experimental

A dry 50 ml flask was charged with benzaldehyde (10 mmol), naphthalen-2-ol (10 mmol) and 3-methylpiperidine (10 mmol). The mixture was stirred at 373 K for 10 h, then ethanol (15 ml) was added. After heating under reflux for 30 min, the precipitate was filtrated off and washed 3 times with ethanol to give the title compound. Single crystals suitable for X-ray analysis were obtained by slow evaporation of a dichloromethane solution.

### Refinement

All H atoms were calculated geometrically, with C—H = 0.93–0.98 Å, O—H= 0.82 Å, and refined as riding with *U*_iso_(H) = 1.2*U*_eq_(C) or 1.2*U*_eq_(C, O) for methyl and hydroxy Y atoms.

### Crystal data

**Table:**

C_23_H_25_NO	*F*(000) = 712
*M**_r_* = 331.44	*D*_x_ = 1.185 Mg m^−^^3^
Monoclinic, *P*2_1_/*n*	Mo *K*α radiation, λ = 0.71073 Å
Hall symbol: -P 2yn	Cell parameters from 2989 reflections
*a* = 9.2138 (13) Å	θ = 2.6–27.9°
*b* = 10.9056 (13) Å	µ = 0.07 mm^−^^1^
*c* = 18.635 (3) Å	*T* = 292 K
β = 97.007 (10)°	Prism, pale yellow
*V* = 1858.5 (5) Å^3^	0.30 × 0.25 × 0.20 mm
*Z* = 4	

### Data collection

**Table:**

Rigaku SCXmini diffractometer	4422 independent reflections
Radiation source: fine-focus sealed tube	2302 reflections with *I* > 2σ(*I*)
graphite	*R*_int_ = 0.067
Detector resolution: 13.6612 pixels mm^-1^	θ_max_ = 27.9°, θ_min_ = 2.9°
ω scans	*h* = −12→12
Absorption correction: multi-scan (*CrystalClear*; Rigaku, 2005)	*k* = −14→14
*T*_min_ = 0.965, *T*_max_ = 0.979	*l* = −24→24
19217 measured reflections	

### Refinement

**Table:**

Refinement on *F*^2^	Primary atom site location: structure-invariant direct methods
Least-squares matrix: full	Secondary atom site location: difference Fourier map
*R*[*F*^2^ > 2σ(*F*^2^)] = 0.061	Hydrogen site location: inferred from neighbouring sites
*wR*(*F*^2^) = 0.155	H-atom parameters constrained
*S* = 0.99	*w* = 1/[σ^2^(*F*_o_^2^) + (0.0693*P*)^2^] where *P* = (*F*_o_^2^ + 2*F*_c_^2^)/3
4422 reflections	(Δ/σ)_max_ < 0.001
228 parameters	Δρ_max_ = 0.15 e Å^−^^3^
0 restraints	Δρ_min_ = −0.16 e Å^−^^3^

### Special details

**Table:**

Geometry. All e.s.d.'s (except the e.s.d. in the dihedral angle between two l.s. planes) are estimated using the full covariance matrix. The cell e.s.d.'s are taken into account individually in the estimation of e.s.d.'s in distances, angles and torsion angles; correlations between e.s.d.'s in cell parameters are only used when they are defined by crystal symmetry. An approximate (isotropic) treatment of cell e.s.d.'s is used for estimating e.s.d.'s involving l.s. planes.
Refinement. Refinement of *F*^2^ against ALL reflections. The weighted *R*-factor *wR* and goodness of fit *S* are based on *F*^2^, conventional *R*-factors *R* are based on *F*, with *F* set to zero for negative *F*^2^. The threshold expression of *F*^2^ > σ(*F*^2^) is used only for calculating *R*-factors(gt) *etc*. and is not relevant to the choice of reflections for refinement. *R*-factors based on *F*^2^ are statistically about twice as large as those based on *F*, and *R*- factors based on ALL data will be even larger.

### Fractional atomic coordinates and isotropic or equivalent isotropic displacement parameters (Å^2^)

**Table:**

	*x*	*y*	*z*	*U*_iso_*/*U*_eq_	
C1	0.67985 (19)	0.98342 (18)	0.20764 (10)	0.0429 (5)	
H1	0.6443	1.0644	0.2203	0.051*	
C2	0.78426 (19)	0.93769 (17)	0.27180 (10)	0.0424 (5)	
C3	0.9016 (2)	0.86366 (18)	0.26209 (11)	0.0473 (5)	
C4	0.9972 (2)	0.8201 (2)	0.32150 (13)	0.0588 (6)	
H4	1.0778	0.7728	0.3138	0.071*	
C5	0.9718 (2)	0.8470 (2)	0.38980 (13)	0.0640 (6)	
H5	1.0349	0.8168	0.4285	0.077*	
C6	0.8527 (2)	0.9195 (2)	0.40364 (11)	0.0560 (6)	
C7	0.7590 (2)	0.96718 (17)	0.34408 (10)	0.0466 (5)	
C8	0.6424 (2)	1.04328 (19)	0.36039 (12)	0.0580 (6)	
H8	0.5790	1.0764	0.3227	0.070*	
C9	0.6211 (3)	1.0690 (2)	0.42986 (13)	0.0739 (7)	
H9	0.5445	1.1201	0.4388	0.089*	
C10	0.7124 (3)	1.0197 (3)	0.48762 (14)	0.0845 (9)	
H10	0.6959	1.0367	0.5349	0.101*	
C11	0.8255 (3)	0.9467 (2)	0.47479 (12)	0.0724 (7)	
H11	0.8863	0.9139	0.5136	0.087*	
C12	0.5482 (2)	0.89924 (19)	0.19333 (10)	0.0466 (5)	
C13	0.4093 (2)	0.9444 (2)	0.19799 (11)	0.0601 (6)	
H13	0.3973	1.0267	0.2090	0.072*	
C14	0.2885 (2)	0.8692 (3)	0.18655 (13)	0.0719 (7)	
H14	0.1959	0.9008	0.1901	0.086*	
C15	0.3047 (3)	0.7479 (3)	0.16990 (13)	0.0713 (7)	
H15	0.2231	0.6972	0.1620	0.086*	
C16	0.4413 (2)	0.7016 (2)	0.16489 (11)	0.0655 (6)	
H16	0.4524	0.6193	0.1536	0.079*	
C17	0.5626 (2)	0.7767 (2)	0.17657 (11)	0.0541 (5)	
H17	0.6550	0.7444	0.1731	0.065*	
C18	0.6619 (2)	1.0167 (2)	0.07506 (10)	0.0580 (6)	
H18A	0.6042	1.0904	0.0784	0.070*	
H18B	0.5954	0.9477	0.0671	0.070*	
C19	0.7514 (2)	1.0277 (2)	0.01231 (11)	0.0680 (7)	
H19A	0.8024	0.9511	0.0068	0.082*	
H19B	0.6864	1.0421	−0.0318	0.082*	
C20	0.8616 (2)	1.1307 (2)	0.02313 (11)	0.0634 (6)	
H20A	0.8106	1.2087	0.0216	0.076*	
H20B	0.9225	1.1298	−0.0158	0.076*	
C21	0.9571 (2)	1.11783 (19)	0.09503 (11)	0.0531 (5)	
H21	1.0152	1.0428	0.0935	0.064*	
C22	0.8604 (2)	1.10436 (19)	0.15507 (11)	0.0509 (5)	
H22A	0.9217	1.0940	0.2008	0.061*	
H22B	0.8038	1.1788	0.1580	0.061*	
C23	1.0612 (3)	1.2248 (2)	0.11110 (14)	0.0819 (8)	
H23A	1.0064	1.2996	0.1113	0.123*	
H23B	1.1262	1.2293	0.0746	0.123*	
H23C	1.1171	1.2132	0.1576	0.123*	
N1	0.75966 (16)	0.99877 (14)	0.14314 (8)	0.0455 (4)	
O1	0.93357 (14)	0.82835 (13)	0.19587 (8)	0.0575 (4)	
H1A	0.8851	0.8690	0.1646	0.086*	

### Atomic displacement parameters (Å^2^)

**Table:**

	*U*^11^	*U*^22^	*U*^33^	*U*^12^	*U*^13^	*U*^23^
C1	0.0391 (10)	0.0487 (11)	0.0419 (11)	0.0029 (9)	0.0094 (8)	−0.0008 (9)
C2	0.0363 (10)	0.0451 (10)	0.0454 (11)	−0.0067 (9)	0.0030 (8)	−0.0013 (9)
C3	0.0389 (11)	0.0505 (12)	0.0522 (13)	−0.0056 (9)	0.0047 (9)	−0.0016 (10)
C4	0.0405 (11)	0.0570 (13)	0.0758 (17)	−0.0008 (10)	−0.0054 (11)	0.0039 (12)
C5	0.0608 (15)	0.0662 (15)	0.0594 (16)	−0.0155 (13)	−0.0149 (11)	0.0122 (12)
C6	0.0570 (13)	0.0584 (14)	0.0507 (13)	−0.0203 (12)	−0.0008 (10)	0.0018 (11)
C7	0.0485 (12)	0.0482 (11)	0.0435 (12)	−0.0139 (10)	0.0070 (9)	−0.0010 (9)
C8	0.0602 (14)	0.0652 (14)	0.0513 (13)	−0.0089 (12)	0.0177 (10)	−0.0074 (11)
C9	0.0778 (17)	0.0849 (18)	0.0635 (17)	−0.0153 (15)	0.0265 (14)	−0.0185 (14)
C10	0.100 (2)	0.105 (2)	0.0508 (16)	−0.0353 (18)	0.0194 (15)	−0.0203 (15)
C11	0.0845 (18)	0.0852 (18)	0.0454 (14)	−0.0310 (16)	−0.0009 (12)	0.0018 (13)
C12	0.0365 (10)	0.0610 (13)	0.0423 (11)	0.0007 (10)	0.0045 (8)	0.0067 (10)
C13	0.0417 (12)	0.0763 (15)	0.0633 (14)	0.0043 (12)	0.0100 (10)	0.0097 (12)
C14	0.0362 (12)	0.109 (2)	0.0708 (16)	−0.0001 (14)	0.0087 (11)	0.0197 (15)
C15	0.0502 (14)	0.095 (2)	0.0660 (15)	−0.0220 (14)	−0.0047 (11)	0.0137 (14)
C16	0.0624 (15)	0.0728 (16)	0.0578 (14)	−0.0103 (13)	−0.0066 (11)	−0.0003 (12)
C17	0.0418 (11)	0.0633 (13)	0.0557 (13)	−0.0011 (11)	0.0001 (9)	−0.0021 (11)
C18	0.0482 (12)	0.0814 (16)	0.0437 (12)	0.0037 (11)	0.0036 (10)	0.0040 (11)
C19	0.0598 (14)	0.1003 (19)	0.0444 (13)	−0.0009 (14)	0.0082 (10)	0.0022 (12)
C20	0.0637 (14)	0.0794 (16)	0.0503 (13)	0.0048 (13)	0.0201 (11)	0.0082 (12)
C21	0.0520 (12)	0.0551 (12)	0.0551 (13)	−0.0024 (10)	0.0184 (10)	−0.0001 (10)
C22	0.0498 (12)	0.0539 (12)	0.0500 (12)	−0.0030 (10)	0.0105 (9)	−0.0017 (10)
C23	0.0856 (18)	0.0819 (17)	0.0834 (18)	−0.0242 (15)	0.0308 (14)	−0.0037 (14)
N1	0.0394 (9)	0.0572 (10)	0.0403 (9)	−0.0036 (8)	0.0065 (7)	−0.0003 (8)
O1	0.0443 (8)	0.0662 (10)	0.0620 (10)	0.0069 (7)	0.0062 (7)	−0.0071 (8)

### Geometric parameters (Å, °)

**Table:**

C1—N1	1.493 (2)	C14—H14	0.9300
C1—C12	1.518 (3)	C15—C16	1.370 (3)
C1—C2	1.524 (3)	C15—H15	0.9300
C1—H1	0.9800	C16—C17	1.381 (3)
C2—C3	1.379 (3)	C16—H16	0.9300
C2—C7	1.431 (3)	C17—H17	0.9300
C3—O1	1.359 (2)	C18—N1	1.477 (2)
C3—C4	1.411 (3)	C18—C19	1.516 (3)
C4—C5	1.354 (3)	C18—H18A	0.9700
C4—H4	0.9300	C18—H18B	0.9700
C5—C6	1.402 (3)	C19—C20	1.510 (3)
C5—H5	0.9300	C19—H19A	0.9700
C6—C11	1.410 (3)	C19—H19B	0.9700
C6—C7	1.419 (3)	C20—C21	1.517 (3)
C7—C8	1.419 (3)	C20—H20A	0.9700
C8—C9	1.362 (3)	C20—H20B	0.9700
C8—H8	0.9300	C21—C23	1.517 (3)
C9—C10	1.391 (4)	C21—C22	1.520 (3)
C9—H9	0.9300	C21—H21	0.9800
C10—C11	1.356 (4)	C22—N1	1.479 (2)
C10—H10	0.9300	C22—H22A	0.9700
C11—H11	0.9300	C22—H22B	0.9700
C12—C17	1.383 (3)	C23—H23A	0.9600
C12—C13	1.384 (3)	C23—H23B	0.9600
C13—C14	1.378 (3)	C23—H23C	0.9600
C13—H13	0.9300	O1—H1A	0.8200
C14—C15	1.371 (3)		
			
N1—C1—C12	112.81 (15)	C14—C15—H15	120.1
N1—C1—C2	110.03 (15)	C15—C16—C17	120.2 (2)
C12—C1—C2	110.74 (15)	C15—C16—H16	119.9
N1—C1—H1	107.7	C17—C16—H16	119.9
C12—C1—H1	107.7	C16—C17—C12	120.7 (2)
C2—C1—H1	107.7	C16—C17—H17	119.6
C3—C2—C7	118.38 (18)	C12—C17—H17	119.6
C3—C2—C1	121.25 (17)	N1—C18—C19	109.91 (16)
C7—C2—C1	120.33 (17)	N1—C18—H18A	109.7
O1—C3—C2	123.06 (18)	C19—C18—H18A	109.7
O1—C3—C4	115.67 (18)	N1—C18—H18B	109.7
C2—C3—C4	121.3 (2)	C19—C18—H18B	109.7
C5—C4—C3	120.1 (2)	H18A—C18—H18B	108.2
C5—C4—H4	120.0	C20—C19—C18	112.10 (19)
C3—C4—H4	120.0	C20—C19—H19A	109.2
C4—C5—C6	121.6 (2)	C18—C19—H19A	109.2
C4—C5—H5	119.2	C20—C19—H19B	109.2
C6—C5—H5	119.2	C18—C19—H19B	109.2
C5—C6—C11	121.6 (2)	H19A—C19—H19B	107.9
C5—C6—C7	118.5 (2)	C19—C20—C21	110.91 (17)
C11—C6—C7	119.9 (2)	C19—C20—H20A	109.5
C6—C7—C8	116.79 (19)	C21—C20—H20A	109.5
C6—C7—C2	120.03 (19)	C19—C20—H20B	109.5
C8—C7—C2	123.18 (19)	C21—C20—H20B	109.5
C9—C8—C7	121.6 (2)	H20A—C20—H20B	108.0
C9—C8—H8	119.2	C20—C21—C23	112.82 (18)
C7—C8—H8	119.2	C20—C21—C22	109.29 (17)
C8—C9—C10	120.8 (3)	C23—C21—C22	110.02 (17)
C8—C9—H9	119.6	C20—C21—H21	108.2
C10—C9—H9	119.6	C23—C21—H21	108.2
C11—C10—C9	119.7 (2)	C22—C21—H21	108.2
C11—C10—H10	120.1	N1—C22—C21	112.21 (16)
C9—C10—H10	120.1	N1—C22—H22A	109.2
C10—C11—C6	121.2 (2)	C21—C22—H22A	109.2
C10—C11—H11	119.4	N1—C22—H22B	109.2
C6—C11—H11	119.4	C21—C22—H22B	109.2
C17—C12—C13	118.2 (2)	H22A—C22—H22B	107.9
C17—C12—C1	121.84 (17)	C21—C23—H23A	109.5
C13—C12—C1	119.93 (19)	C21—C23—H23B	109.5
C14—C13—C12	121.0 (2)	H23A—C23—H23B	109.5
C14—C13—H13	119.5	C21—C23—H23C	109.5
C12—C13—H13	119.5	H23A—C23—H23C	109.5
C15—C14—C13	120.1 (2)	H23B—C23—H23C	109.5
C15—C14—H14	120.0	C18—N1—C22	109.40 (15)
C13—C14—H14	120.0	C18—N1—C1	113.43 (14)
C16—C15—C14	119.8 (2)	C22—N1—C1	109.15 (14)
C16—C15—H15	120.1	C3—O1—H1A	109.5
			
N1—C1—C2—C3	−31.0 (2)	C7—C6—C11—C10	−1.1 (3)
C12—C1—C2—C3	94.4 (2)	N1—C1—C12—C17	63.6 (2)
N1—C1—C2—C7	151.44 (16)	C2—C1—C12—C17	−60.2 (2)
C12—C1—C2—C7	−83.2 (2)	N1—C1—C12—C13	−117.4 (2)
C7—C2—C3—O1	178.73 (17)	C2—C1—C12—C13	118.81 (19)
C1—C2—C3—O1	1.1 (3)	C17—C12—C13—C14	0.3 (3)
C7—C2—C3—C4	−1.6 (3)	C1—C12—C13—C14	−178.80 (18)
C1—C2—C3—C4	−179.21 (17)	C12—C13—C14—C15	−0.3 (3)
O1—C3—C4—C5	−177.90 (18)	C13—C14—C15—C16	0.2 (4)
C2—C3—C4—C5	2.4 (3)	C14—C15—C16—C17	0.0 (3)
C3—C4—C5—C6	−0.9 (3)	C15—C16—C17—C12	−0.1 (3)
C4—C5—C6—C11	179.4 (2)	C13—C12—C17—C16	0.0 (3)
C4—C5—C6—C7	−1.3 (3)	C1—C12—C17—C16	179.00 (18)
C5—C6—C7—C8	−178.04 (18)	N1—C18—C19—C20	−57.0 (3)
C11—C6—C7—C8	1.2 (3)	C18—C19—C20—C21	53.8 (3)
C5—C6—C7—C2	2.0 (3)	C19—C20—C21—C23	−175.32 (19)
C11—C6—C7—C2	−178.67 (17)	C19—C20—C21—C22	−52.6 (2)
C3—C2—C7—C6	−0.6 (3)	C20—C21—C22—N1	57.4 (2)
C1—C2—C7—C6	177.01 (17)	C23—C21—C22—N1	−178.25 (18)
C3—C2—C7—C8	179.47 (17)	C19—C18—N1—C22	59.5 (2)
C1—C2—C7—C8	−2.9 (3)	C19—C18—N1—C1	−178.37 (18)
C6—C7—C8—C9	−0.2 (3)	C21—C22—N1—C18	−61.3 (2)
C2—C7—C8—C9	179.68 (19)	C21—C22—N1—C1	174.09 (16)
C7—C8—C9—C10	−0.9 (3)	C12—C1—N1—C18	43.3 (2)
C8—C9—C10—C11	1.1 (4)	C2—C1—N1—C18	167.50 (16)
C9—C10—C11—C6	0.0 (4)	C12—C1—N1—C22	165.56 (15)
C5—C6—C11—C10	178.1 (2)	C2—C1—N1—C22	−70.23 (19)

### Hydrogen-bond geometry (Å, °)

**Table:**

*D*—H···*A*	*D*—H	H···*A*	*D*···*A*	*D*—H···*A*
O1—H1A···N1	0.82	1.84	2.570 (2)	148
